# Anxiolytic-Like Effects of Compound Zhi Zhu Xiang in Rats

**DOI:** 10.1155/2012/701289

**Published:** 2012-05-29

**Authors:** Yan-Li Wang, Jin-Li Shi, Liu Yong, Zhao Ren, Yu-Jing Zhai, Jian-You Guo

**Affiliations:** ^1^School of Chinese Materia Medica, Beijing University of Chinese Medicine, Beijing 100102, China; ^2^Pharmaceutical Factory, Yunnan Institute of Material Medical, Yunnan 650111, China; ^3^Key Laboratory of Mental Health, Institute of Psychology, Chinese Academy of Sciences, Beijing 100101, China

## Abstract

The purpose of this study was to determine whether compound zhi zhu xiang (CZZX) exerts anxiolytic-like effects in rats. The animals were orally administered CZZX (0.75, 1.5, and 3 g/kg daily) for 10 days and tested in the elevated plus maze (EPM), Vogel conflict test (VCT), and open field. Repeated treatment with CZZX (3 g/kg/day, p.o.) significantly increased the percentage of both entries into and time spent on the open arms of the EPM compared with saline controls. In the VCT, repeated treatment with CZZX (1.5 and 3 g/kg/day, p.o.) significantly increased the number of punished licks. The drug did not change the total entries into the open arms of the EPM or interfere with water consumption or nociceptive threshold, discarding potential confounding factors in the two tests. In the open field, locomotion was not reduced, discarding the possible sedative effect of CZZX. In the binding assay, the binding of [^3^H] Ro 15-1788 (flumazenil) to the benzodiazepine binding site in washed crude synaptosomal membranes from rat cerebral cortex was affected by CZZX. These data indicate an anxiolytic-like profile of action for CZZX without sedative side effects, and this activity may be mediated by benzodiazepine binding site modulation at *γ*-aminobutyric acid-A receptors.

## 1. Introduction

Anxiety is a widespread incapacitating psychiatric ailment that imposes a substantial health burden on society. Benzodiazepines (BZDs) are considered safe drugs and widely prescribed for their anxiolytic, muscle relaxant, sedative-hypnotic, and anticonvulsant actions [1]. However, they may produce side effects, such as sedation and myorelaxation, that are considered unwanted effects of anxiolytic drugs [2]. Therefore, the search for safe and effective agents has continued.

Over the past decade, herbal medicines have received increasing attention from the psychiatry research community. This is largely because many herbal preparations have been found to have beneficial effects for various psychiatric conditions in experimental animal and clinical studies [3–6]. In Western societies, natural remedies for anxiety disorders, such as valerian (*Valeriana officinalis*), St. John's wort (*Hypericum perforatum*), passion flower (*Passiflora incarnata*), hops (*Humulus lupulus*), and kava kava (*Piper methysticum*), are readily available [7]. Several herbal mixtures also have anxiolytic-like effects in rodent models of anxiety (e.g., Yokukansan [8] and Xiao-Tan-Jie-Yu-Fang [9]). Moreover, substantial evidence from our laboratories indicates that herbal medicines are beneficial in preventing mental illness, such as vanadium-enriched *Cordyceps sinensis *[5], gan mai da zao decoction [10], and Sini tang [6]. 

Compound zhi zhu xiang (CZZX) originated from a clinical experimental prescription, and a clinical study showed that it is an effective and well-tolerated antianxiety prescription [11]. Among the constituents of CZZX, *Valeriana jatamansi* Jones accounts for the largest proportion and plays the major therapeutic role. It acts as an “emperor herb” according to traditional Chinese medicine formulation theory. The plant in India is widely known for its use in the treatment of anxiety, insomnia, epilepsy, failing reflexes, hysteria, neurosis, and sciatica [12]. In some countries, it is used as an important sedative in herbal medicine. Moreover, large-scale studies also demonstrated that extracts of *Valeriana officinalis* L. s.l. exert anxiolytic effects [13, 14]. *Ziziphus jujuba* Mill. and *Albizia julibrissin *Durazz. play auxiliary roles as “minister herbs” in the CZZX formulation because of their effects on insomnia. In animal studies, *Ziziphus jujuba *Mill. had anxiolytic-like effects at lower doses and sedative effects at higher doses in the black and white test and elevated plus maze [15]. *Juncus effusus* L. plays an assisting role in CZZX as an “assistant and messenger herb.” Clinical studies in China revealed that it can regulate different mental disorders, such as anxiety, improve sleep, and improve gastrointestinal function. However, animal studies have not demonstrated the ability of CZZX to mediate anxiety.

The purpose of the present study was to determine whether CZZX exerts anxiolytic-like effects in rats. The animals were tested in the elevated plus maze, Vogel conflict test (VCT), and open field. The binding affinity and efficacy of CZZX at the *γ*-aminobutyric acid-A- (GABA_A_-) BZD receptor were determined to reveal the potential mechanisms that underlie the selective behavioral profile of CZZX.

## 2. Material and Methods

### 2.1. Animals

Male Sprague-Dawley rats (160–180 g) were obtained from the Laboratory Animal Center of the Academy of Military Medical Sciences and used for this study. The animals were housed five per cage under controlled temperature (22 ± 1°C) and a 12 h/12 h light/dark cycle (lights on at 07:00 AM) with free access to food and water. The experimenter handled the animals daily to acclimate them to the manipulation. The animals were used only once and for only one experiment. The experimental procedures were approved by the Institutional Animal Care and Use Committee of the Institute of Psychology of the Chinese Academy of Sciences and in accordance with the National Institutes of Health Guide for Care and Use of Laboratory Animals.

### 2.2. Drugs and Treatment

Four crude drugs (*Valeriana jatamansi*, *Ziziphus jujuba*, *Albizia julibrissin*, and *Juncus effusus*) were purchased from the Medicinal Materials Company of Hebei Anguo and authenticated by Jin-Li Shi, Department of Botany, Beijing Chinese Medical University, based on their micro- and macroscopic characteristics. The quality of these crude drugs was controlled and processed according to the Chinese Pharmacopoeia. Drug samples were collected as voucher specimens and kept with the records. The 35% ethanolic v/v extract of *Valeriana jatamansi* (200 g/2000 mL; reflux, 2 h × 3), water extracts of *Ziziphus jujuba* and *Albizia julibrissin* (200 g/2000 mL; decoction, 3 h × 2), and 95% ethanolic v/v extract of *Juncus effusus* (20 g/300 mL; reflux, 1 h × 2) were filtered and dried under reduced pressure at a temperature <60°C. They were then mixed at the ratio stated in the prescription.

The doses are expressed in terms of the dried weight of the CZZX extract per unit body weight of the experimental animals (g/kg). Diazepam was chosen as a positive control drug. For oral (p.o.) administration for 10 days, CZZX and diazepam were dissolved in saline. Each drug was administered in a volume of 1 mL/100 g body weight. Control animals received vehicle (saline) only. In this study, the rats were administered CZZX or DZP 60 and 30 min before the test, respectively. The opioid agonist morphine hydrochloride was obtained from Sigma (St. Louis, MO, USA). Morphine was administered at a dose of 5 mg/kg in a sterile 0.9% NaCl solution via intraperitoneal (i.p.) injection 30 min before the nociceptive test. All of the animal tests were performed on the 10th day of treatment.

### 2.3. Elevated Plus Maze

Anxiolytic activity was measured using the elevated plus maze [16]. The maze consisted of two open arms (50.8 cm × 10.2 cm × 1.3 cm) and two closed arms (50.8 cm × 10.2 cm × 40.6 cm) that extended from a central platform (10.2 cm × 10.2 cm). The maze was elevated to a height of 72.4 cm above the floor. The entire maze was constructed of clear Plexiglas. On the test days, the animals were transported to the elevated plus maze room and left undisturbed in a neutral box for 5 min prior to testing. Immediately after this period, each rat was placed on the central square facing an open arm and allowed to freely explore the maze for 5 min. Arm entries were defined as the entry of all four paws into an arm. A computer recorded the time spent on and number of entries into the open and closed arms by means of infrared photocells. The percentage of open arm entries (100 × open/total entries) was calculated for each animal. The apparatus was wiped clean with water and dried after each subject.

### 2.4. Vogel Conflict Test

The VCT was performed in a Plexiglas box (29 cm × 29 cm × 26 cm) with a stainless steel grid floor. The metallic spout of a drinking bottle that contained water projected into the box. The simultaneous contact of the animal with the spout and the grid floor closed an electrical circuit controlled by a sensor, producing seven pulses of water per second whenever the animal was in contact with both components. Each pulse was considered a lick. After every 20 licks, the animal received a 0.5 mA footshock for 2 s. The sensor recorded the total number of licks and shocks delivered during the test period. The entire apparatus was located inside a sound-attenuated cage.

### 2.5. Water Consumption Evaluation

The apparatus was the same as the one used in the VCT described above, but the electric shock-delivering system was rendered inoperative.

### 2.6. Acute Thermal Pain

A radiant heat apparatus was used to induce acute pain. The thermal thresholds of the rats hindpaws were measured. Each rat was placed in a plastic chamber on a glass floor, under which a radiant heat apparatus (100 W projector lamp) was located. A beam of light through a 4 mm diameter hole in the apparatus was focused on the plantar surface of the left hindpaw. The paw withdrawal latency (PWL) was defined as the time between the light onset and paw withdrawal and adjusted to approximately 8 s to record the baseline. A cutoff time of 22 s was used to avoid tissue damage. Four trials spaced at least 5 min apart were conducted with each hindpaw. The last three trials were averaged to provide the mean latency.

### 2.7. Open Field Test

The open field was a 180 cm diameter cylinder with 60 cm high walls. The center of the bottom of the apparatus had a 50 cm diameter section. The rats were placed into the field at the same point against the wall and allowed to freely explore the apparatus for 10 min. The total path length was recorded by an automatic video tracking system. Grooming time, the number of rearings, and the number of defecations were also recorded. After each trial, the apparatus was wiped clean with a 10% ethanol solution.

### 2.8. Biochemical Assay

#### 2.8.1. Tissue Preparation

The preparation was performed at 4°C. The cerebral cortex from four rats was homogenized for 5 s in 20 mL of 50 mM Tris-citrate (pH 7.1) using an Ultra-Turrax. The suspension was centrifuged at 27,000 ×g for 15 min, and the pellet was washed three times with buffer. The washed pellet was homogenized in 20 mL buffer. The suspension was incubated in a water bath at 37°C for 30 min to remove endogenous GABA and then centrifuged for 10 min at 27,000 ×g. The final pellet was resuspended in 30 mL buffer and stored in aliquots at −20°C.

#### 2.8.2. [^3^H] Ro 15-1788 (Flumazenil) Binding Assay

The binding assay was performed according to a previously described method [17] with modifications. The membrane preparation was thawed and washed with 20 mL of 50 mM Tris-citrate (pH 7.1) at 4°C. The suspension was then centrifuged at 27,000 ×g for 10 min at 4°C. The pellet was resuspended in 50 mM Tris-citrate (pH 7.1) with 2 mg of the original tissue per milliliter of buffer and then used for the binding assay. The membrane suspension (500 *μ*L) was then added to 25 *μ*L of test solution (CZZX extract/standard/blank) and 25 *μ*L flumazenil (Ro 15-1788, 78 Ci/mmol; Perkin-Elmer Life Sciences), mixed, and incubated for 40 min in an ice bath. Nonspecific binding was determined using diazepam (1 *μ*M, final concentration in assay) added to separate samples. After incubation, 5 mL of ice-cold buffer was added to the samples, and the mixture was poured directly onto Adventic glass fiber filters (GC-50) under suction and immediately washed with 5 mL of ice-cold buffer. The amount of radioactivity was determined by conventional liquid scintillation counting. Specific binding was calculated as total binding minus nonspecific binding. All of the experiments were performed in triplicate.

### 2.9. Statistical Analysis

The data are expressed as mean ± SEM. The data from the elevated plus maze, VCT, acute thermal pain assessment, and open field were analyzed using one-way analysis of variance (ANOVA) followed by Dunnett's test across the five groups. Probability levels less than 0.05 were considered statistically significant.

## 3. Results

### 3.1. Elevated Plus Maze

**Figure 1:**
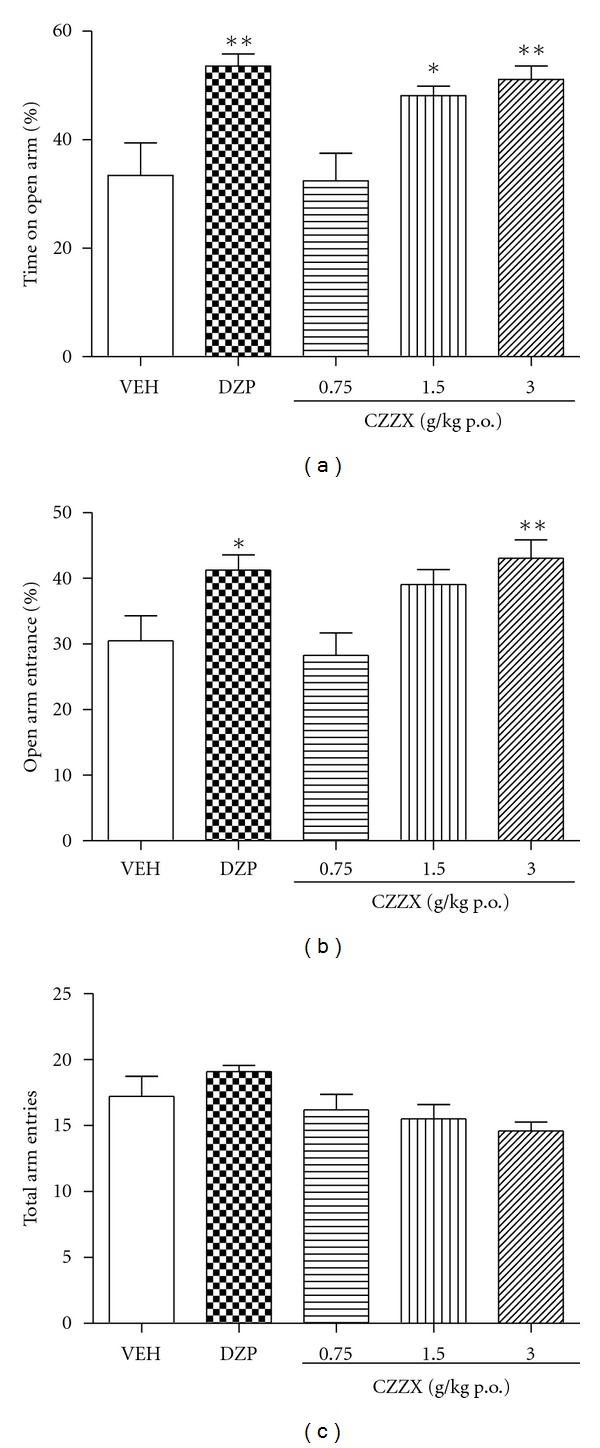
Behavioural performance of rat registered in a 5 min session in the elevated plus maze performed 1 h after the injection of vehicle (VEH, p.o.) and CZZX (0.75, 1.5 and 3 g/kg, p.o.) or 0.5 h after the injection of diazepam (DZP, 1 mg/kg, p.o.). (a) Percentage of the number of entries into the open arm, (b) percentage of time spent into the open arms and (c) total arm entries. Columns represent the means ± SEM, *n* = 9-10 rats. *P < 0.05, **P < 0.01 compared to the control group.

The one-way ANOVA indicated significant differences among groups in the time spent in the open arms of the elevated plus maze (*F*_4,44_ = 6.72, *P* < 0.01; Figure 1(a)) and percentage of open arm entries (*F*_4,44_ = 5.08, *P* < 0.01; Figure 1(b)). CZZX at a dose of 3 g/kg produced anxiolytic-like effects, reflected by an increase in the percentage of open arm entries (*P* < 0.05). Doses of 1.5 and 3 g/kg also increased the time spent in the open arms of the maze (*P* < 0.05 and *P* < 0.01, resp.). No differences were observed in total arm entries (*F*_4,44_ = 0.88, *P* > 0.05; Figure 1(c)).

### 3.2. Vogel Conflict Test

**Figure 2:**
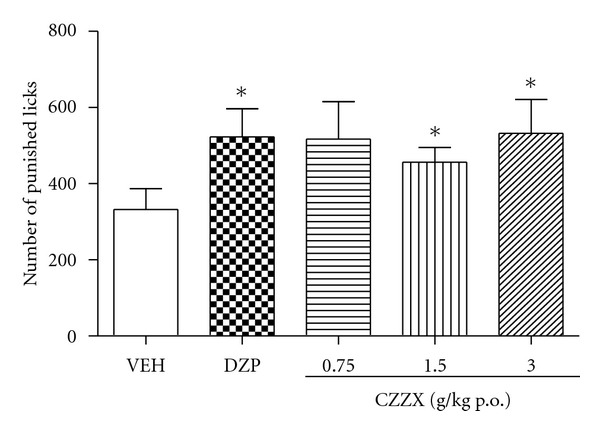
Nubmer of punished licks registered in a 5 min session in the vogel conflict test performed 1 h after the injection of vehicle (VEH, p.o.) and CZZX (0.75, 1.5 and 3 g/kg, p.o.) or 0.5 h after the injection of diazepam (DZP, 1 mg/kg, p.o.). Columns represent the means ± SEM, *n* = 9-10 rats. *P < 0.05 compared to the control group.

To confirm the anxiolytic-like effects of CZZX, we tested an independent group of rats in the VCT. In this experiment, 50 animals were used. Consistent with the previous experiment, the one-way ANOVA revealed significant variance among the five groups (*F*_4,44_ = 2.03, *P* < 0.01). Doses of 1.5 and 3 g/kg significantly increased the number of punished licks compared with controls (both *P* < 0.05; Figure 2).

### 3.3. Water Consumption

**Figure 3:**
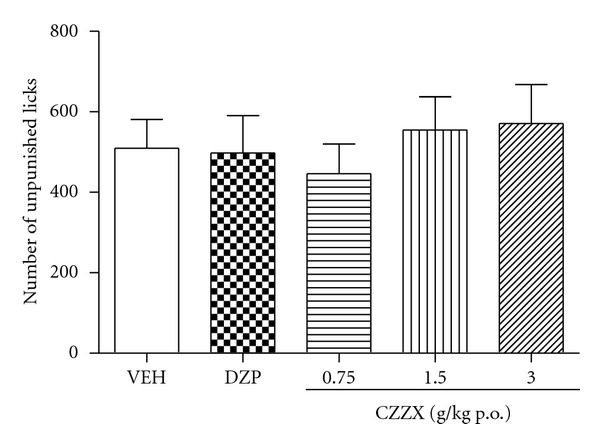
Effects of CZZX on the number of unpunished licks of rats that had been water-deprived for 48 h. Columns represent the means ± SEM, *n* = 10 rats.

Additional experiments were performed to test whether CZZX increases water consumption and nociceptive responses, which are potential confounding factors in the VCT. However, no differences in the number of unpunished licks were observed among the five groups (*F*_4,45_ = 0.35, *P* > 0.05; Figure 3).

### 3.4. Acute Thermal Pain

**Figure 4:**
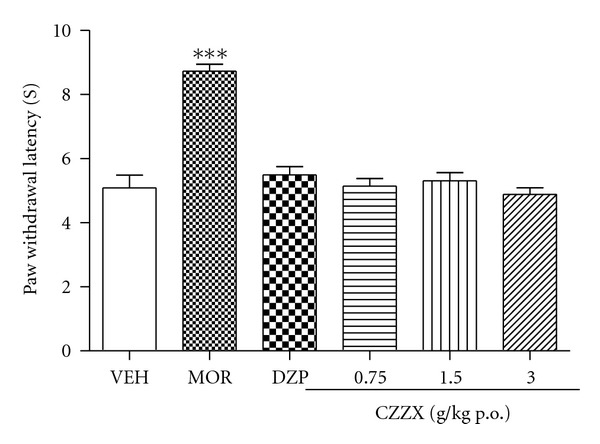
Effects of CZZX on the nociceptive thermal thresholds. Columns represent the means ± SEM, *n* = 10 rats. ***P < 0.01 compared to the control group.

We also investigated the effects of saline, CZZX, and morphine on acute thermal nociceptive thresholds measured with noxious radiant heat in normal rats. The one-way ANOVA revealed a significant effect of treatment (*F*_5,54_ = 29.67, *P* < 0.05; Figure 4). Morphine significantly increased the PWL compared with the saline control (*P* < 0.001). In contrast to the analgesic effect of morphine, CZZX did not have any effect on the PWL induced by the noxious heat stimulus (*P* > 0.05).

### 3.5. Open Field Test

**Figure 5:**
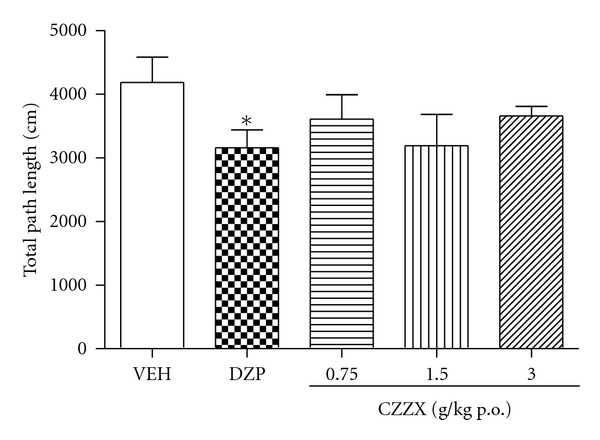
Effect of CZZX on the exploratory behavior of rats in the open field test. Columns represent the means ± SEM, *n* = 10 rats. *P < 0.05 compared to the control group.

No difference in total path length was observed among the five groups (*F*_4,44_ = 1.98, *P* > 0.05; Figure 5), with no difference in emotional behavior (i.e., rearing, grooming, and defecation; data not shown).

### 3.6. Binding Affinity of CZZX to GABA_A_-BZD Receptors

**Figure 6:**
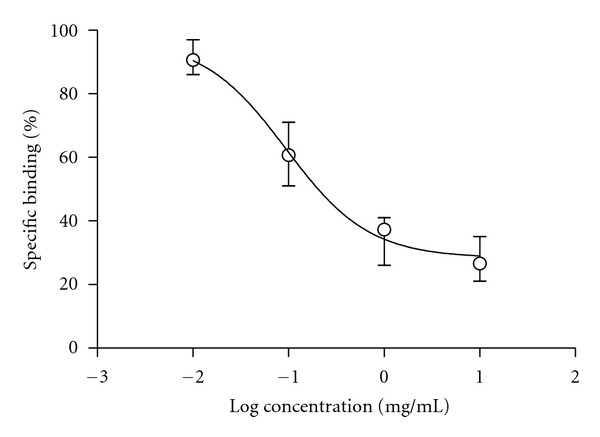
Activity of CZZX in the [^3^H] Ro 15-1788 (flumazenil) binding assay. Data represent mean ± SEM of four independent experiments each performed in triplicate.

In the GABA_A_-BZD binding assay, CZZX was able to displace flumazenil binding. Therefore, it may inhibit the binding of the specific radioligand [^3^H] Ro 15-1788 to the BZD receptor (Figure 6).

## 4. Discussion

The present study sought to analyze the behavioral effects of CZZX, which is used to treat “nervous diseases” in traditional Chinese medicine [11]. The results showed that CZZX exhibited anxiolytic-like activity and did not induce sedative side effects. The binding assay suggested that the anxiolytic effects of CZZX may be attributable to the modulation of GABA_A_ receptors. Altogether, our results lend support to the traditional use of CZZX in Chinese folk medicine. The present findings may also provide important leads for the development of potent and selective anxiolytic agents.

When the animals were treated with the higher doses of CZZX (1.5 and 3 g/kg) for 10 days, anxiety-like behavior in the elevated plus maze was significantly attenuated, although the low dose (0.75 g/kg) had no effects on the open-arm indices, suggesting that CZZX possesses anxiolytic effects in the elevated plus maze paradigm. Behavior in the elevated plus maze is related to the natural aversion that rodents have for elevated and open spaces. Anxiolytic drugs shift the behavioral response toward exploration of the open arms [18]. The main potential confounding factors in this model are changes in basal locomotor activity, which can be inferred from the total number of entries. CZZX did not alter total arm exploration in the elevated plus maze, suggesting that this drug induces specific anxiolytic-like effects.

To further strengthen these data, we tested the anxiolytic-like effects of these treatments in the VCT, which involves the suppression of punished responses. The behavioral suppression induced by shocks in the VCT is attenuated by anxiolytic drugs [19–21]. One drawback of this paradigm, however, is that drugs that induce antinociceptive effects or increase water consumption may yield false-positive results. The doses of 1.5 and 3 g/kg CZZX increased the number of punished licks (i.e., induced anxiolytic-like effects), but none of the doses altered the number of unpunished licks. Thus, this is unlikely to be a confounding factor in the assessment of anxiolytic-like effects. Moreover, CZZX was ineffective in the acute thermal pain test, excluding changes in nociceptive threshold as confounding factors.

The results obtained in the open field test showed that chronic CZZX treatment did not affect the distance traveled or emotional behavior (i.e., rearing, grooming, and defecation), suggesting that this compound may not produce undesirable sedative side effects. These results indicate that repeated treatment with CZZX may significantly improve anxiety-like behavior without producing sedative side effects. Many individual herbal preparations of CZZX and their major constituents, including *Valeriana jatamansi *[12], *Ziziphus jujuba *[22], *Albizia julibrissin *[23], and *Medulla junci* [24], exert anxiolytic or sedative effects in various animal models. Given that the anxiolytic-like effects of CZZX observed in the present study were generated from the effects of individual herbs, one may expect that CZZX has superior effects in ameliorating anxiety compared with individual herbal preparations.

In traditional Chinese prescriptions, herbs are usually mixed before extraction. However, the four herbs contained in the formula are all sensitive to water temperature. Some scientific references and experimental evaluations of the isolation of the active principles of each plant have been provided [25], and we extracted the four herbs individually using different solvents and then mixed the extracts according to the ratio of crude medicines in the CZZX prescription. Preliminary pharmacodynamic studies are being performed in our laboratory to investigate anxiolytic-like effects in mice. We found that these four herbs that were extracted individually exhibited higher potency in anxiolytic tests than the four herbs mixed before extraction (data not shown). Therefore, these four herbs in the formula were extracted individually in the present study.

GABA is the most important inhibitory neurotransmitter in the human central nervous system. Compelling evidence suggests an imbalance between excitatory and inhibitory neurotransmitters in the pathophysiology of convulsions, anxiety, and sleep [26]. The GABA_A_ receptor system is the main fast-acting inhibitory neurotransmitter system in the brain and the pharmacological target for many drugs used clinically to treat anxiety disorders and epilepsy. GABA_A_ receptors are heteromeric GABA-gated chloride channels. The BZD site on GABA_A_ receptors modulates the inhibitory effects of GABA [27]. Benzodiazepine site agonists, such as diazepam, increase the GABA-induced chloride channel opening frequency, exerting anxiolytic, anticonvulsant, muscle relaxant, sedative-hypnotic, and cognition-impairing effects [28] and rendering these agonists the most important GABA_A_ receptor-modulating drugs in clinical use. For this reason, discovering plants that enhance GABA affinity for the GABA_A_ receptor is important.

In the present study, diazepam was used as a positive control. As expected, it increased activity in the open arms of the elevated plus maze and number of punished licks in the VCT, confirming its anxiolytic actions. Many benzodiazepines and related compounds that bind to receptors in the central nervous system have been identified in certain plant extracts [29, 30]. In the search for the underlying mechanism of the anxiolytic-like effect of CZZX, we performed a complementary assay to investigate whether the effects of CZZX were attributable to its action at GABA_A_ receptors. The binding studies were conducted using the GABA antagonist [^3^H] Ro 15-1788 (flumazenil). In the GABA_A_-BZD binding assay, CZZX displaced [^3^H] Ro 15-1788 to brain synaptosomal membranes, indicating that CZZX had effective concentration-dependent binding activity and suggesting central BZD-like activity. However, the EC_50_ for CZZX was approximately 0.5 mg/mL, and further pharmacokinetic and pharmacodynamic studies are needed in the future.

In summary, the present study showed that CZZX elicited strong effects on anxiety-like behavior, likely mediated by its BZD-like activity. The extent of anxiolytic activity was comparable to the effects of diazepam, but CZZX may be devoid of undesirable side effects, such as sedation. Therefore, CZZX is a promising candidate for the treatment of anxiety-like disorders. Studies are being performed in our laboratories to isolate the active principles of CZZX and determine the specific effects of CZZX on the central nervous system and its underlying mechanism of action.
